# What Pelvic Floor Muscle Training Load is Optimal in Minimizing Urine Loss in Women with Stress Urinary Incontinence? A Systematic Review and Meta-Analysis

**DOI:** 10.3390/ijerph16224358

**Published:** 2019-11-08

**Authors:** Esther García-Sánchez, Vicente Ávila-Gandía, Javier López-Román, Alejandro Martínez-Rodríguez, Jacobo Á. Rubio-Arias

**Affiliations:** 1 Department of Exercise Physiology, Universidad Católica de Murcia, 30107 Murcia, Spain; egsanchez@ucam.edu (E.G.-S.); vavila@ucam.edu (V.Á.-G.); jlroman@ucam.edu (J.L.-R.); 2 Fundación para la Formación e Investigación Sanitarias de la Región de Murcia, 30003 Murcia, Spain; 3 Health Sciences PhD program, Universidad Católica de Murcia, 30107 Murcia, Spain; 4 Department of Analytical Chemistry, Nutrition and Food Science, Faculty of Science, Alicante University, 03690 Alicante, Spain; amartinezrodriguez@gcloud.ua.es; 5 Faculty of Sports, UCAM, Universidad Católica de Murcia, 30107 Murcia, Spain; 6 Department of Health and Human Performance, Faculty of Physical Activity and Sport Science-INEF, Universidad Politécnica de Madrid, 28040 Madrid, Spain

**Keywords:** exercise, incontinence, training, training load, women’s health issues

## Abstract

Pelvic floor muscle training is commonly used for urine loss. However, research studies have not determined which training load is the most effective for women with stress urinary incontinence (SUI). Moreover, none of the previous reviews or studies have described the total effectiveness of pelvic floor muscle training (PFMT) with an objective test such as the pad test. The objectives were to analyze the effectiveness of pelvic floor muscle training in women with SUI and to determine which training load produces the greatest adaptations for decreasing urine loss. The search was conducted in three databases (PubMed, Web of Science and Cochrane), for randomized controlled trials (RCTs) that evaluated the effects of PFMT. Studies were included if they met the following criteria: participants were women; were older than 18; had SUI; were treated with PFMT; and the assessments of the effects were measured with a pad test. Finally, 10 articles (293 women) analyzed the pad test in women with SUI who performed PFMT. The meta-analysis showed that PFMT, independent of the protocol used in the study, resulted in decreased urine loss in women suffering from SUI. However, for large effects, the program should last 6–12 weeks, with >3 sessions/week and a length of session <45 min.

## 1. Introduction

The incidence data on urinary incontinence (UI) in the female population is very dissimilar, but according to International Continence Society (ICS) data, 10% of the female population suffers urine leakage weekly and between 25% and 45% of the female population suffers urine leakage occasionally. Hunskaar et al., in 2004 [1], analyzed the prevalence of urinary incontinence in women from four European countries and showed that stress UI (SUI) was the most common type overall, although the relative prevalence of mixed symptoms increased with age, with SUI being the most common in the population aged >55. Nonetheless, not only was age associated with UI, but there were other factors associated with it as well. Thus, Hunskaar, in 2008 [2], after a systematic review, concluded that overweight and obesity were important risk factors for UI. Other risk factors highlighted by the ICS were parity, pregnancy and mode of delivery [3,4], ethnicity and race [5], hysterectomy [6], menopausal replacement therapy [7], diets, such as coffee intake [8], socioeconomic status [3], smoking [9], physical activity [10] and other comorbidities (i.e., depression, physical impairment, diabetes…).

Presently, there are different tests for the diagnosis and monitoring of UI. Among these are the evaluation of symptoms in the lower urinary tract [11,12], the questionnaires on quality of life, with the International Consultation Incontinence Questionnaire and King’s Health Questionnaire being the most commonly used [13], physical exploration (electromyography, dynamometry, echography and magnetic resonance) [14], as well as a bladder diary [15]. In addition to these, the pad test, a non-invasive diagnostic tool for UI, is also found. A recent review showed that the pad test was a valid, objective, accessible and cheap diagnostic tool for the monitoring and evaluation of urine loss, although its use was not frequent in the urology field. The pad test consists of using a pad to control the loss of urine, thus weighing the previous pad and then weighing it again one or twenty-four hours later. [16]. Because quality of life is considered as an important factor by women [17], treatment decisions should not be based solely on a patient’s subjective perception of the severity of UI [18]. Moreover, decreases in urine leakages (pad test) altered the quality of life (incontinence-specific quality of life (QOL) questionnaire) after a conservative treatment (floor muscle exercises).

Thus far, several studies [19,20,21] have distinguished between conservative treatment and surgical treatment. A conservative treatment is usually used for SUI and several types exist, such as pelvic floor muscle training (PFMT), biofeedback physical therapy, vaginal cones or electro-stimulation. However, electro-stimulation and/or biofeedback should be used for women who cannot actively contract pelvic floor muscles (PFM) in order to improve motivation and adherence to therapy [22]. The main objective of these treatments is, on the one hand, to facilitate urinary continence by strengthening the PFM. Such treatments imply an intense contraction of the pubococcygeus muscle without using the muscles of the abdomen or buttocks [23]. A systematic review and meta-analysis showed a positive tendency of the success of PFMT for women suffering from SUI.

In fact, any PFMT program must include specific characteristics, that have not been specified and constitute an important clinical uncertainty [24]. Dumoulin et al., in 2007 [25], showed that there was a significant reduction of urine loss with a training program. Fillmore et al., in 2011, showed that women also had significant improvements in their UI symptoms after 12 weeks, although the characteristics of the training program were different. In this study, the women performed exercises two times a week, having to perform slow contractions and fast contractions [26]. There is evidence that the PMFT is an effective treatment for reducing urine leakage [27,28,29,30,31,32]. Nevertheless, there was a wide range of training protocols available for PFMT and systematic reviews and meta-analysis studies to date have not yet determined which characteristics of training (conservative treatments—PMFT) were the most effective for urine leakage (pad test) in women with SUI. Therefore, the aims of the present systematic review and meta-analysis were (1) to analyze the effectiveness of pelvic floor muscle exercises (PFMEs) using the pad test in women with stress urinary incontinence (SUI) and (2) to determine which pelvic floor muscle training characteristics (length of the program, frequency, duration, exercises) produced the greatest adaptations for decreasing urine loss.

## 2. Materials and Methods

### 2.1. Data Source and Searches

The search was conducted in three databases from inception through 1 January and no restrictions by dates were applied. The following electronic databases were searched: PubMed, Web of Science and Cochrane (Cochrane Database of Systematic Reviews, Cochrane Central Register of Controlled Trials and Cochrane Library). The database searches were performed by two authors (VAG and EGS). The search keywords and strategy used were: (“woman” OR “female”) AND (“urinary incontinence” OR “stress urinary incontinence”) AND (“pelvic floor muscle exercises” OR “pelvic floor muscle treatment” OR “pelvic floor muscle training”) for each database.

### 2.2. Selection Criteria

Research studies were included if they were randomized controlled trials (RCTs), SUI treatments, and clinical treatments published in English. The studies were chosen if (1) participants were women; (2) were 18 years or older; (3) had SUI (involuntary leakage of urine on effort or exertion); (4) had a urodynamic diagnosis of SUI or symptoms of SUI (5) were treated with PFMT; (6) the ones performing PFMT were not treated with any other kind of treatment (electrical stimulations, bladder training, pharmaceuticals, surgery, advice on fluid management, etc.) and (7) the assessments of the effects were measured with a pad test; (8) were original research studies (published papers). Research studies were excluded if they included: (1) pregnant participants; (2) post-partum; (3) UI prevalence; (4) prevention; and (5) abstracts, conference summaries, and theses.

### 2.3. Study Selection and Data Extraction

The eligibility criteria were pre-defined by the authors. The studies containing randomized controlled trials (RCTs) were included, and some research restrictions of language, date and literature were applied. Nonetheless, review articles and case reports were not included in the analysis. Two different authors (VAG and EGS) conducted tabulated research separately, and the chosen index had identical default outcomes. Discrepancies in methodology, gathering of articles and statistical analysis were solved via consensus among all the authors.

The data collection process was performed by one reviewer (EGS) and supervised by another (JARA), and the following information was extracted from each article chosen: (1) author and year of publication; (2) sample characteristics: size, age, disease and medical status; (3) study design; (4) interventions: control and experimental group; (5) training characteristics: type (pelvic floor training group, Balloon, Biofeedback, Floor, Group, Individual, Pelvic, Supervised, Training, Vaginal Cone, Supine, Vertical,), frequency, duration, session length and number of sessions; (6) pad test characteristics: units (mg or mL) and type (1 h or 24 h assessment); and (7) standardized mean, standard deviation, or raw data for effect size calculation. The reviewers did not show disparities in data abstraction. All data items were listed and defined in a specific template. The reviewers (EGS and JARA) used standardized templates for data extraction and included data for each study or group.

### 2.4. Study Quality (Risk of Bias Assessment)

The methodological quality of the randomized trial studies was assessed according to the PEDro scale [33,34,35], using the following criteria: (1) the eligibility criteria were specified; (2) the participants were randomly assigned to the groups (in a crossover study, the participants were randomly assigned to treatment groups); (3) the assignment was hidden; (4) the groups were similar at the start of the study in relation to the most important prognostic indicators; (5) all the participants were blinded; (6) there was blinding of all the therapists that administered the therapy; (7) there was blinding of the evaluators that measured at least one key result; (8) measurements of key results were obtained from more than 85% of the participants that were initially assigned to the groups; (9) all the participants whose results were available received treatment or were part of the control group according to which they were assigned to, or when this was not the case, the data for at least one key result were analyzed by ‘intention-to-treat’; (10) the results of the statistical comparisons between groups were reported for at least one key result; and (11) the study provided both specific and variability measurements for at least one key result. Due to the characteristics of the studies, points 4 and 5 were not evaluated. The publication bias was evaluated through an asymmetry test as estimated from a funnel plot (Figure 2). In addition, Egger’s test was used to assess publication bias.

### 2.5. Data Synthesis and Statistics Analysis

This search followed the Preferred Reported Items for Systematic Reviews and Meta-Analysis (PRISMA) guideline [36] recommendations. The meta-analysis and statistical analyses were carried out using the Review Manager Software (RevMan 5.2, Cochrane Collaboration, Oxford, UK) and the Comprehensive Meta-Analysis software (version 2, Biostat Inc, Englewood, NJ, USA). For each trial, the effect size (ES) of the intervention was calculated by subtracting mL/g of urine loss before and after the intervention. For controlled trials, the ES of PFMT was also calculated in terms of the difference of urine loss before and after the intervention among women performing PFMT and the control group.

Each difference in means was weighted according to the inverse variance method [37]. Urine loss was evaluated using the same method (pad test). However, the population characteristics and those from the training program used in each of the studies were taken into account (Table 2). The difference in means was standardized by dividing values with the corresponding standard deviation. The standardized mean differences (SMD) from each trial were collected in a random effects model [38]. The ES was calculated by using the Cohen guideline. 

$$S=\frac{Mpre-Mpos}{Spre}\left(1\;-\;\frac{3}{4n\;-\;5}\right)$$

According to the Cohen guidelines, threshold values for ES were ≤0.4 (small), 0.5–0.7 (moderate), ≥0.8 (large). [39] Heterogeneity between the studies was evaluated through *I^2^* statistics. A scale of magnitude was implemented for the interpretation of heterogeneity of the results, where ≤25% was assessed as low magnitude, ≤50% was medium magnitude and ≥75% was high magnitude [40]. The analysis between the subgroups in relation to the population characteristics and the training performed was conducted through dichotomous or continuous variables that could have an influence on the improved results after RCT. In most cases, the median was used as the cut-off value of the variables studied, allowing for pair-wise comparison. However, in specific cases, the cut-off was established in an arbitrary manner. The changes in those factors that could potentially be influential were expressed and analyzed as values prior to the intervention minus the values found after the intervention. Publication bias was considered with the funnel plot, and Egger’s test was used to assess publication bias. A *p*-value below 0.05 was considered to be statistically significant.

## 3. Results

### 3.1. Characteristics of Included Studies

**Figure 1:**
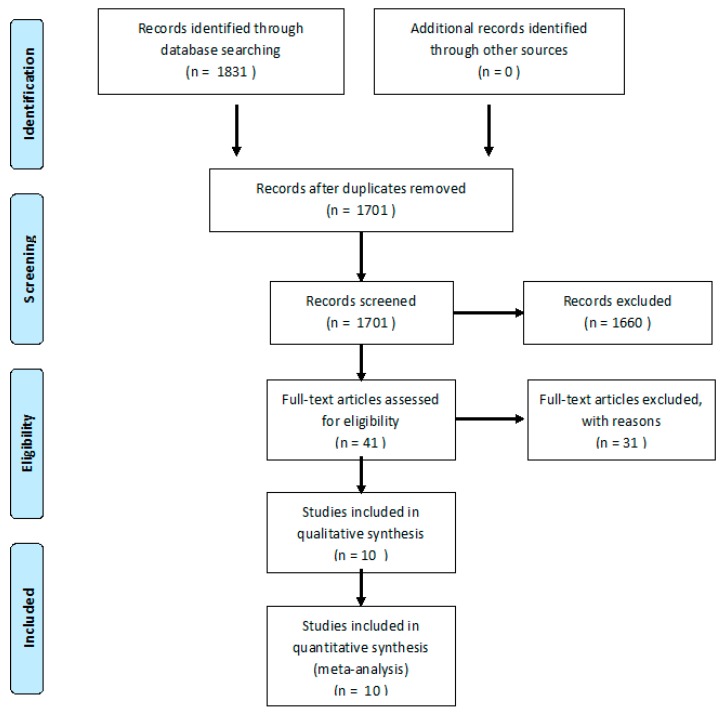
Flow diagram of the process of study selection.

From a total of 1831 articles, 1701 were analyzed after removing duplicates. In total, 293 women were evaluated for SUI, with PFMT or control protocol. After the evaluation of 1701 abstracts from primary sources, 1660 were excluded and 41 were assessed as full texts. From these, 31 studies were excluded. Thus, 10 RCTs (*n* = 293 women) were included [41,42,43,44,45,46,47,48,49,50]. The flow diagram showing the process of study selection is shown in Figure 1.

The main characteristics and properties of the included studies are summarized in Table 1 Lastly, in this review, there were 10 RCT-eligible studies.

The characteristics of the PFMT intervention in different groups and urinary incontinence assessment of the studies included in the meta-analysis are presented in Table 2.

### 3.2. Risk of Bias Assessment

**Figure 2:**
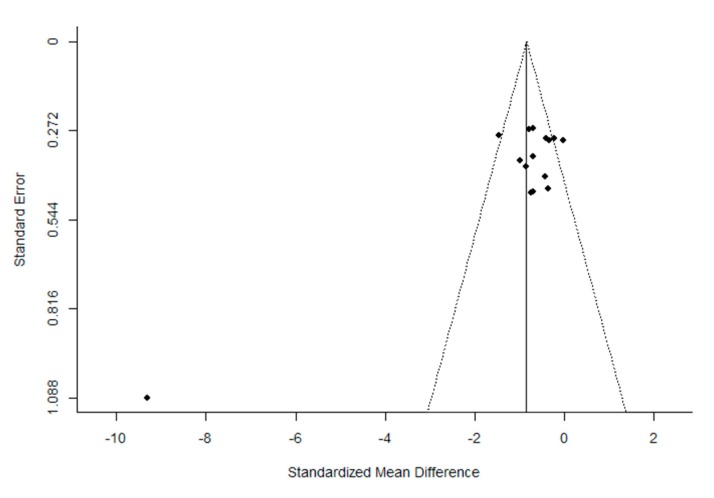
Funnel plot of the meta-analysis of the published studies. Each plotted point represents the standard error (SE) and the standardized mean difference (SMD) between post-intervention intervention urinary loss in women who received PFMT.

**Figure 3:**
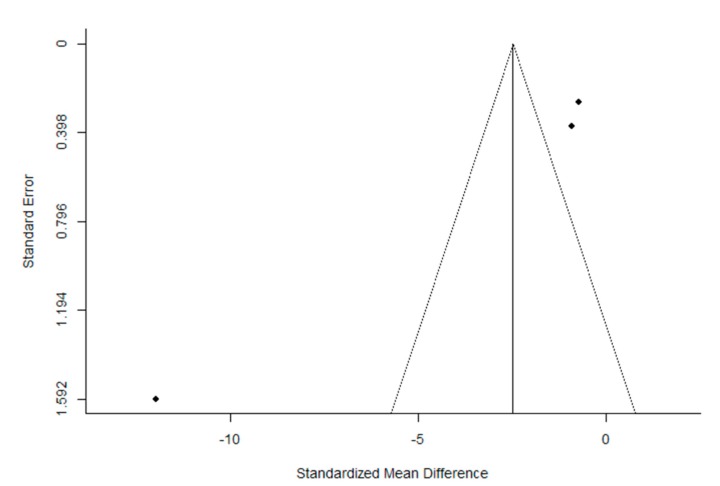
Funnel plot of the meta-analysis of the published studies. Each plotted point represents the standard error (SE) and the standardized mean difference (SMD) between post-intervention and pre-intervention urinary loss in women who received PFMT for a single study.

The quality of the trials, according to the PEDro scale, was found to be high. The mean (± SD) score was 7.2 ± 1.4 out of a possible 11 points. After the evaluation of potential bias, the funnel plot for the SMD between post- and pre-intervention urinary loss in PFMT female participants was notably asymmetrical, suggesting a significant publication bias (Figure 2 and Figure 3). Similar results were obtained for the evaluation of the potential bias of the SMD in post-intervention urinary loss between the PFMT and control women groups. In addition, Egger’s test results showed significant heterogeneity (Post- vs. pre-intervention: *z* = −5.950, *p* < 1.001; PFMT vs CG: −6.636, *p* < 0.001).

### 3.3. Meta-Analysis

**Figure 4:**
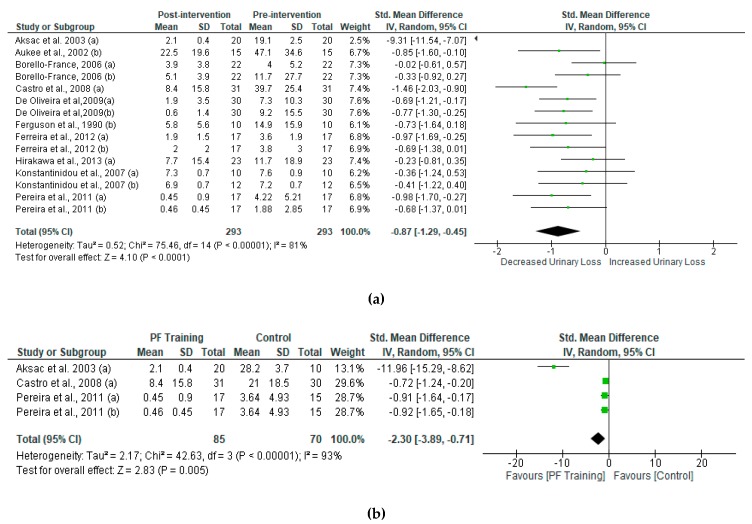
Standardized mean difference (SMD) (**a**) between post- and pre-intervention urinary loss in women who received PFMT and (**b**) post-intervention intervention urinary loss in women who received PFMT. Squares represent the SMD for each trial. Diamonds represent the pooled SMD across trials.

The overall effect of PMFT led to a significant decrease in urine loss. To compare differences between post- and pre-intervention urinary incontinence in women who received PFMT, our study analyzed the standard mean difference of each group and compared them (Figure 4a). Women who received PFMT showed a reduction of urinary loss. The results of the SMD in post-intervention urinary incontinence between PFMT and control women are shown in Figure 4b. We found significant changes in urinary incontinence in women who received PFMT.

#### Sub-Group Analysis

Within the subgroup analysis (Table 3), PFMT had a favorable effect on urinary loss. However, the results indicated that there were no significant differences according to the groups on the subgroup analyses, except for the frequency of the intervention, showing improvement when frequency was equal to or greater than 3 days per week (range 3–7). Regarding the comparison between PFMT and PFMT with training equipment (e.g., vaginal cone), the results confirmed that groups trained with equipment or accessories showed significant improvements compared to the PFMT group without the use of equipment. Likewise, women trained with equipment showed greater improvements as compared to biofeedback. As for the other variables, no differences among subgroups were found. In addition, the women included in the studies who scored higher on the PEDro scale after training showed less urine leakage.

## 4. Discussion

The main objective of this systematic review with meta-analysis was to analyze published randomized clinical trials that investigated the effects of PFMT through specific relaxation and tightening muscle exercises on women suffering from SUI using the pad test as an objective measurement to assess the pathology and thus determine the main characteristics that a PFMT protocol should have and also to determine in which type of women it could provide better results. To date, the effect of this type of training on quality of life has been shown. However, no previous systematic reviews or meta-analyses have been found that examine the effect of PMFT on an objective test. The meta-analysis suggests that PFMT was an effective treatment for SUI, i.e., the women suffering from SUI and who underwent treatment, showed a decrease in urine loss. In this sense, one major find was that women with SUI, regardless of age, took advantage of PFMT and that this treatment was equally effective in women aged under 53 [41,42,43,45,46,47,50] and in those over 53 years old [43,44,47,48,49]. Similarly, no significant differences were found regarding BMI. There was UI improvement both in women who had a BMI of less than or equal to 26 [44,45,48,49] and in those women with a BMI higher than 26 [45,47,49]. Therefore, the PFMT was deemed effective regardless of age and BMI, which are considered to be the main risk factors for SUI.

Regarding exercise characteristics, our analysis showed that no differences were observed between subgroups when divided according to training duration (weeks or sessions per week) or session duration. However, women who trained for a longer period of time (>12 weeks or ≥24 sessions) with shorter sessions (10–45 min) tend to achieve a greater decrease in urine loss. Also, training frequency showed significant differences in favor of those studies that applied the treatment 3 to 7 days a week [41,42,44,47,50], compared to studies that included training programs with a frequency of less than three sessions per week [43,44,45,48,49]. Therefore, these results suggest that it is more important to accumulate a greater number of shorter sessions than a smaller number of longer sessions. A possible explanation for this might be that the training programs that lasted 8 weeks [41,51] were insufficient for reaching muscle hypertrophy, as the improvement may be due to learning factors [31]—the reason for this is likely due to a greater number of short sessions resulting in associated motor learning retention and so a greater building of gains. As far as the number of contractions, these should not exceed 200 per day [31]. According to results obtained in this meta-analysis, some studies, such as the one by Miller et al. in 1998 [52], which declared that the application of a one-week program resulted in an improvement in the coordination between a maximum voluntary pelvic floor contraction and an increase in intra-abdominal pressure, and the study by Bø et al. in 1999 [53] that recommended doing 8 to 12 maximum voluntary PF contractions, holding them for approximately 6 to 8 s, and 3 or 4 fast contractions at the end, pausing for 6 s between contractions, performing it 3 times per day. Finally, and considering these research studies, we may confirm that PFM contraction and the moment in which it is performed are key factors for the maintenance of continence. In this sense, the authors suggest a training program of 6 weeks minimum for reaching an improvement. These contractions should be carried out by combining slow contractions or holding for 5, 6 to 10 s, with rapid contractions lasting 1, 2 and 3 s. The recovery time between contractions may range from 1 to 12 s, depending on the number of contractions performed. The series should not exceed nine per session, and recovery between them should be 1 to 3 min, with these variables to be used incrementally. In this sense, to increase the intensity, the number of contractions, duration of contraction or number of fast contractions should be increased. Lastly, the use of vaginal cones of different weights and biofeedback could be added as a complement, as they could assist the work performed by the PF musculature.

As for the equipment used for the training sessions, significant differences were found in favor of training with equipment or accessories [44,46]. A greater effectiveness in treatment with biofeedback was observed. Along this line, the effectiveness of treatment with equipment (vaginal cones) and the effectiveness of treatment with biofeedback were compared, resulting in similar benefits for women using vaginal cones versus those following a biofeedback treatment. However, it would be advisable to implement training with material as it improves control of information on contractions (biofeedback) [42] and increased stimulus overload (vaginal cones). [44].

A decrease in urine loss (according to the pad test) may be justified by a significant reduction of the internal surface of the elevator muscle of the anus after having performed PFM exercises, which leads to an increase in passive stiffness of this muscle, which in turn is an indicator of PF muscle tone status. As previously stated by Dumoulin et al. in 2007 [25], there was a significant difference in the resting pressure of a woman’s PFM, and according to Griffin et al. in 1994 [54], as well as greater stability in the urethra both when relaxing and during effort, as stated in Balmforth et al. in 2004 [55], after 14 weeks of supervised training. Therefore, there is ample evidence that supports the conclusion that PFMT improves PF muscle tone and automatic muscle motor control while preventing a decrease in PF strength during increases in intra-abdominal pressure, thereby preventing loss of urine [56].

There are several limitations to the analysis that must be considered. The first limitation of this study was the low number of clinical trials included in the meta-analysis. Other limitations include the significant publication risk of bias results in the meta-analysis. In this sense, we found that the included literature heterogeneity was high—81% when comparing the effects of post vs. pre-treatment in the PFMT group and 99% when comparing the effects of the PFMT group vs. control group. Differences in population characteristics (e.g., vaginal delivery, race or city in which the study was conducted), and training protocols (e.g., duration of contractions), could be affecting our results, masking the PFMT effect due to the large number of training-related variables involved. In addition, the significant differences between the groups analyzed with the PEDro scale suggest that future studies should meet a minimum set of recommendations in order to better determine the effect of PFMT on women with UI. This reflects a limitation and the results of this meta-analysis should be interpreted with caution.

## 5. Conclusions

Pelvic floor muscle training may be an effective therapy for reducing urine loss in women with stress urinary incontinence. In addition, the results suggest that PFMT using short sessions (10–45 min) and with a frequency of 3 and 7 days per week might evoke the greatest changes in women with UI.

## Figures and Tables

**Table 1:** Characteristics of the included studies in the meta-analysis.

Study, Year of Publication	Level of Physical Activity	Vaginal Delivery	Country of the Study	Type of Training	C	PFT *n*	♀ (%)	Age (Years)	BMI (cm/kg^2^)
Aksac, 2003 [41]	Sedentary	2.8 ± 0.5	Turkey	PFT (a)	10	20	100	52.5 ± 7.9	N/A
		3.5 ± 1.1		PFTBi (b)		20		51.6 ± 5.8	N/A
Aukee et al. 2002 [42]	Sedentary	2.2 (0–5)	Finland	Bi (a)	-	15	100	35–61	21–36
		3.2 (0–7)		PFT (b)		15		31–69	21–36
Borello-France et al. 2006 [43]	Sedentary	no	EEUU	SuPFT (a)	-	22	100	51.7 ± 8.9	N/A
		no		SuVPFT (b)		22		53.6 ± 8.1	N/A
Castro et al. 2008 [44]	Sedentary	2.8 ± 1.9	Brazil	PFT (a)	30	31	100	56.2 ± 12.5	25.9 ± 5.0
		3.5 ± 2.6		ES (b)		30		55.2 ± 12.8	21.9 ± 3.9
		3.0 ± 2.1		VC (c)		27		52.6 ± 11.2	24.1 ±4.6
De Oliveira et al. 2009 [45]	Sedentary	2.7 ± 2.4	Brazil	GPFT (a)	-	30	100	51.6 ± 9.6	26.8 ± 4.5
		2.6 ± 2.1		IPFT (b)		30		50.3 ± 8.7	25.5 ± 4.7
Ferguson et al., 1990 [46]	Sedentary	1.7 ± 1.4	EEUU	PFTIB (a)	-	10	100	37.1 ± 6.4	N/A
		1.9 ± 1.0		PFT (b)		10		35.8 ± 4.6	N/A
Ferreira et al., 2012 [47]	Sedentary	2.47 ± 1.2	Portugal	PFTS (a)	-	17	100	50.7 ± 9.3	29.2 ± 4.7
		2.24 ± 1.0		PFT (b)		17		53.9 ± 8.7	27.2 ± 4.1
Hirakawa et al., 2013 [48]	Sedentary	2.1 ± 0.6	Japan	PFT (a)	-	23	100	58.3 ± 11.2	22.5 ± 2.3
		2.2 ± 0.7		PFTBi (b)		23		55.3 ± 9.8	23.9 ± 4.2
Pereira et al., 2011 [49]	Sedentary	1.46 ± 1.5	Brazil	GPFT (a)	15	17	100	60.2 ± 8.2	26.03 ± 3.6
		1.26 ± 1.3		PFT (b)		17		60.6 ± 12.6	26.26 ± 2.79
Konstantinidou, 2007 [50]	Sedentary	no	Greece	PFT (a)	-	10	100	47.8 ± 7.5	N/A
		no		PFTS (b)		12			

Data are the mean, mean ± SD or *n*. ^a^ All characteristics refer to the CT group. C, control group; PFT, pelvic floor training group. Ba, Balloon; Bi, Biofeedback; F, Floor; G, Group; I, Individual; P, Pelvic; S, Supervised; T, Training; VC, Vaginal Cone; Su, Supine; V, Vertical; N/A, Not appear; ES, Electrical Stimulation.

**Table 2:** Characteristics of pelvic floor training intervention and urinary loss assessment of the studies included in the meta-analysis.

	Type Training	Frequency (Week^−1^)	Session Length (min)	Duration (Weeks)	Number of Sessions	Units	Pad Test Type
Aksac et al. 2003 [41]	PFT (a)	3	7.5–15	8	24	g	1 h
	PFTBi (b)	3	20	8	24	g	1 h
Aukee et al. 2002 [42]	Bi (a)	5	20	12	60	g	24 h
	PFT (b)	5	20	12	60	g	24 h
Borello-France et al. 2006 [43]	SuPFT (a)	2	N/A	9–12	18–24	g	1 h
	SuVPFT (b)	2	N/A	9–12	18–24	g	1 h
Castro et al. 2008 [44]	PFT (a)	3	45	24	72	g	1 h
	ES (b)	3	20	24	72	ml	1 h
	VC (c)	3	20	24	72	g	1 h
De Oliveira et al. 2009 [45]	GPFT (a)	2	45	12	24	g	1 h
	IPFT (b)	2	30	12	24	g	1 h
Ferguson et al., 1990 [46]	PFTIB (a)	N/A	10	6	N/A	g	24 h
	PFT (b)	N/A	10	6	N/A	g	24 h
Ferreira et al., 2012 [47]	PFTS (a)	7	N/A	24	168	g	1 h
	PFT (b)	7	N/A	24	168	g	1 h
Hirakawa et al., 2013 [48]	PFT (a)	2	N/A	12	24	g	1 h
	PFTBi (b)	2	N/A	12	24	g	1 h
Pereira et al., 2011 [49]	GPFT (a)	2	60	6	12	g	1 h
	PFT (b)	2	60	6	12	g	1 h
Konstantinidou, 2007 [50]	PFT (a)	7	N/A	12	84	g	24 h
	PFTS (b)	7	N/A	12	84	g	24 h

Data are the mean range. g, grams; mL, milliliters. Ba, Balloon; Bi, Biofeedback; F, Floor; G, Group; I, Individual; P, Pelvic; S, Supervised; T, Training; VC, Vaginal Cone; Su, Supine; V, Vertical; N/A, Not appear; ES, Electrical Stimulation.

**Table 3:** Subgroup analyses assessing potential moderating factors for urinary loss in PFT studies included in the meta-analysis.

Sub-Group	Number ^a^	Study References	ES	Urinary Loss			
				SMD (95% CI)	*I* ^2^	*P*	*P_Difference_*
**Number of participants**							
*n* (15–27)							
≥20	7	Aksac [41] a; Borello-France [43] a, b; Castro [44] a; De Oliveira [45] a, b; Hirakawa [48] a	1.3	−1.24 (−2.07, −0.41)	92	<0.05	0.26
<20	8	Aukee [42] b; Ferguson [45] b; Ferreira [46] a, b; Konstantinidou [50] a, b; Pereira [49] a, b	0.6	−0.74 (−1.03, −0.45)	0	<0.05	
**Age (35–60.6)**							
≥53 y.o.	6	Borello-France [43] b; Castro [44] a; Ferreira [47] b; Hirakawa [48] a; Pereira [49] a, b	0.6	−0.73 (−1.12, −0.33)	57	<0.05	0.37
<53 y.o.	9	Aksac [41] a, Aukee [42] b; Borello-France [43] a; De Oliveira [45] a, b; Ferguson [46] b; Ferreira [47] a, Konstantinidou [50] a, b	1.1	−0.87 (−1.29, −0.40)	80	<0.05	
**BMI (21–36)**							
>26 kg/m^2^	4	De Oliveira [45] a; Ferreira [47] a, b; Pereira [49] b	0.6	−0.74 (−1.06, −0.42)	0	<0.05	0.71
≤26 kg/m^2^	4	Castro [44] a; De Oliveira [45] b; Hirakawa [48] a; Pereira [49] a	0.7	−0.86 (−1.37, −0.34)	67	<0.05	
**Number of sessions**							
≥24 sessions	11	Aksac [41] a, Aukee [42] b; Borello-France [43] a, b; Castro [44] a; De Oliveira [45] a, b; Ferreira [47] a, b, Konstantinidou [50] a, b	1.1	−1.01 (−1.56, −0.45)	86	<0.05	0.22
<24 sessions	4	Ferguson [46] b; Hirakawa [48] a; Pereira [49] a, b	0.5	−0.60 (−0.95, −0.25)	0	<0.05	
**Duration**							
>12 weeks	3	Castro [44] a; Ferreira [47] a, b	0.9	−1.08 (−1.55, −0.61)	34	<0.05	0.51
≤12 weeks	12	Aksac [41] a, Aukee [42] b; Borello-France [43] a, b; De Oliveira [45] a, b; Ferguson [46] b; Hirakawa [48] a; Pereira [49] a, b, Konstantinidou [50] a, b	0.9	−0.85 (−1.35, −0.34)	84	<0.05	
**PFT frequency**							
≥3 days/week	7	Aksac [41] a, Aukee [42] b; Castro [44] a; Ferreira [47] a, b, Konstantinidou [50] a, b	1.5	−1.62 (−2.68, −0.57)	91	<.05	0.05
<3 days/week	7	Borello-France [43] a, b; De Oliveira [45] a, b; Ferguson [46] b; Hirakawa [48] a; Pereira [49] a, b	0.4	−0.53 (−0.75, −0.31)	83	<0.05	
**PFT session length**							
≥45min	4	Castro [44] a, De Oliveira [45] a; Pereira [49] a, b	0.7	−0.96 (−1.35, −0.58)	36	<0.05	0.12
<45min	4	Aksac [41] a, Aukee [42] b; De Oliveira [45] b; Ferguson [46] b	2.0	−2.48 (−4.33, −0.63)	94	<0.05	
**Material**							
No	15	Aksac [41] a, Aukee [42] b; Borello-France 43a, b; De Oliveira [45] a, b; Ferguson [46] b; Hirakawa [48] a; Pereira [49] a, b; Castro [44] a; Ferreira [47] a, b, Konstantinidou [50] a, b	0.9	−0.87 (−1.29, −0.45)	81	<0.05	0.22
Yes	3	Castro [44] b, c; Ferguson [46] a	0.9	−1.27 (−1.75, −0.79)	35	<0.05	
**Biofeedback**							
No	12	Aksac [41] a, Aukee [42] b; Borello-France [43] a, b; De Oliveira [45] a, b; Castro [44] a; Ferguson [46] b; Ferreira [47] a, b; Hirakawa [48] a; Konstantinidou [50] a, b Pereira [49] a, b	0.9	−0.87 (−1.29, −0.45)	81	<0.05	0.04
Yes	3	Aksac [41] b; Aukee [42] a; Hirakawa [48] b	3.8	−1.07 (−1.56, −0.59)	97	<0.05	
Material	3	Castro [44] b, c; Ferguson [46] a	0.9	−1.27 (−1.75, −0.79)	35	<0.05	0.07
Biofeedback	3	Aksac [41] b; Aukee [42] a; Hirakawa [48] b	3.8	−1.07 (−1.56, −0.59)	97	<0.05	
**Pad test**							
1 h	9	Aksac [41] a, Borello-France [43] a, b; Castro [44] a; Ferreira [47] a, b; Hirakawa [48] a; Pereira [49] a, b	1.2	−1.15 (−1.86, −0.45)	89	<0.05	0.12
24 h	4	Aukee [42] b; Ferguson [46] b, Konstantinidou [50] a, b	0.5	−0.53 (−0.87, −0.19)	0	<0.05	
**Methodological quality**							
PEDro scale							
>7 points	3	Aksac [41] a, Castro [44] a; Ferguson [46] b	2.7	−3.53 (−6.42, −0.64)	96	<0.05	0.04
≤7 points	12	Aukee [42] b; Borello-France [43] a, b; De Oliveira [45] a, b; Ferreira [47] a, b, Hirakawa [48] a; Konstantinidou [50] a, b Pereira [49] a, b	0.5	−0.57 (−0.76, −0.38)	0	<0.05	

^a^ Number of PFMT into this study references. Certain enrolled studies were not included because the value used for subgroup analysis was not reported in them. SMD, standardized mean difference; *I^2^*, heterogeneity; test for overall effect; Difference, test for subgroup differences; ES: Effect Size; y.o., years old.
